# Transmantle sign-like calcified radial lesion on CT serves as a potential imaging feature for epileptogenic foci in tuberous sclerosis complex: a Case Report

**DOI:** 10.3389/fradi.2026.1719889

**Published:** 2026-03-12

**Authors:** Zhongke Wang, Xingye Liu, Kaixuan Huang, Chunqing Zhang, Hui Yang, Xiaolin Yang, Shiyong Liu

**Affiliations:** 1 Comprehensive Epilepsy Center, Department of Neurosurgery, Xinqiao Hospital, Army Military Medical University, Chongqing, China; 2 Department of Neurosurgery, Armed Police Hospital of Chongqing, Chongqing, China; 3 Chongqing Institute for Brain and Intelligence, Guangyang Bay Laboratory, Chongqing, China

**Keywords:** drug-resistant epilepsy, imaging feature, neurosurgery, transmantle sign, tuberous sclerosis complex

## Abstract

The tuberous sclerosis complex (TSC) is an important cause of drug-resistant epilepsy (DRE) in children. According to international TSC diagnostic criteria, multiple cortical tubers are a key driver of DRE in these patients. Surgical resection of epileptogenic (epi) tubers remains an effective treatment for TSC-related DRE, and precise preoperative identification of these tubers is critical for favorable surgical outcomes. We report the case of a 2-year-old girl with TSC who presented for epilepsy surgery evaluation. She had a 1.5-year history of DRE and was unresponsive to multiple antiepileptic therapies. During preoperative assessment, conventional MRI failed to detect clear cortical tubers. However, CT imaging revealed rare bilateral hyperdense transmantle sign (TMS)-like lesions in central brain regions, which are usually associated with focal cortical dysplasia type IIb (FCD IIb) on MRI. Scalp electroencephalogram (EEG) and stereoelectroencephalogram (SEEG) monitoring confirmed that the seizures originated from the CT calcified radial lesions resembling TMS, which were subsequently resected. Neuropathological examination of the resected tissue revealed balloon cells and dysmorphic neurons, consistent with epi tubers. A 3-year postoperative follow-up confirmed that these CT calcified radial lesions resembling TMS were epi tubers. Notably, only 5%–10% of TSC cases show atypical cortical tubers on MRI and traditionally identified via metabolic abnormalities on magnetic resonance spectroscopy (MRS) or hypometabolic changes on positron emission tomography (PET). To our knowledge, no previous TSC case with atypical cortical tubers on MRI has been reported to exhibit CT calcified radial lesions resembling TMS. This case highlights the clinical value of CT-specific features in identifying epi tubers, especially when cortical tubers are atypical on conventional MRI.

## 1 Introduction

Tuberous sclerosis complex (TSC) is a multisystem autosomal dominant disorder that affects approximately 1 in 6,000 live births. Epilepsy, the most common neurological manifestation, occurs in 80%–90% of patients with TSC. Approximately 60% of patients with TSC fail to achieve adequate seizure control with antiepileptic drugs alone and progress to drug-resistant epilepsy (DRE) (1, 2). Our previous nationwide multicenter study identified surgical resection of epileptogenic (epi) tubers as the most effective intervention for DRE in TSC patients (3).

Multiple cortical tubers are a characteristic feature of patients with TSC. The updated international diagnostic criteria for TSC have replaced “cortical dysplasia” with “multiple cortical tubers and/or radial migration lines”, underscoring the pivotal role of multiple cortical tubers in TSC-related epilepsy (4). Precise preoperative localization of epi foci among multiple cortical tubers is critical for determining resection strategies, surgical outcomes, and prognosis in TSC-related epilepsy, but it remains a major clinical challenge (5). Non-invasive neuroimaging methods, including computed tomography (CT), magnetic resonance imaging (MRI), and ^18^F-fluorodeoxyglucose positron emission tomography (^18^F-FDG PET), are increasingly used to identify epi tubers using imaging markers (6, 7).

MRI, particularly on T2-weighted fluid-attenuated inversion recovery (FLAIR) sequences, is the conventional standard modality for identifying cortical tubers. Cortical tubers typically appear as hyperintensities on T2-FLAIR images, which aid in delineating tuber extent and supporting preoperative assessment. However, approximately 5%–10% of patients with TSC have atypical cortical tubers on MRI (8, 9). In such cases, metabolic abnormalities detected by magnetic resonance spectroscopy (MRS) or hypometabolic changes detected by positron emission tomography (PET) can be used to identify those atypical cortical tubers (10, 11). Our previous studies have shown that cortical tubers with T2-FLAIR hyperintensity and hyperdense calcifications on CT have a high risk of epileptogenicity. However, the potential value of CT hyperdense calcifications for identifying epi tubers that are atypical on MRI remains unclear.

Here, we present a rare case of a TSC patient with epi tubers that were atypical on MRI but identifiable on CT. Cranial CT revealed two conical hyperdense calcified foci, located in the bilateral central region. The morphology of these foci is analogous to the transmantle sign (TMS) typically observed on MRI, manifesting as goblet-like hyperdense shadows in the white matter that gradually taper from the gray-white matter junction toward the lateral ventricles. However, no obvious abnormalities corresponding to these foci were detected on MRI. Notably, the results of scalp electroencephalogram (EEG), stereoelectroencephalogram (SEEG) and neuropathological examination confirmed that the CT calcified radial lesions resembling TMS were epi tubers. This distinct imaging finding carries important implications for localizing epi tubers in patients with TSC, especially in those with cortical tubers that are difficult to identify on conventional MRI.

## 2 Case description

**Figure 1:**
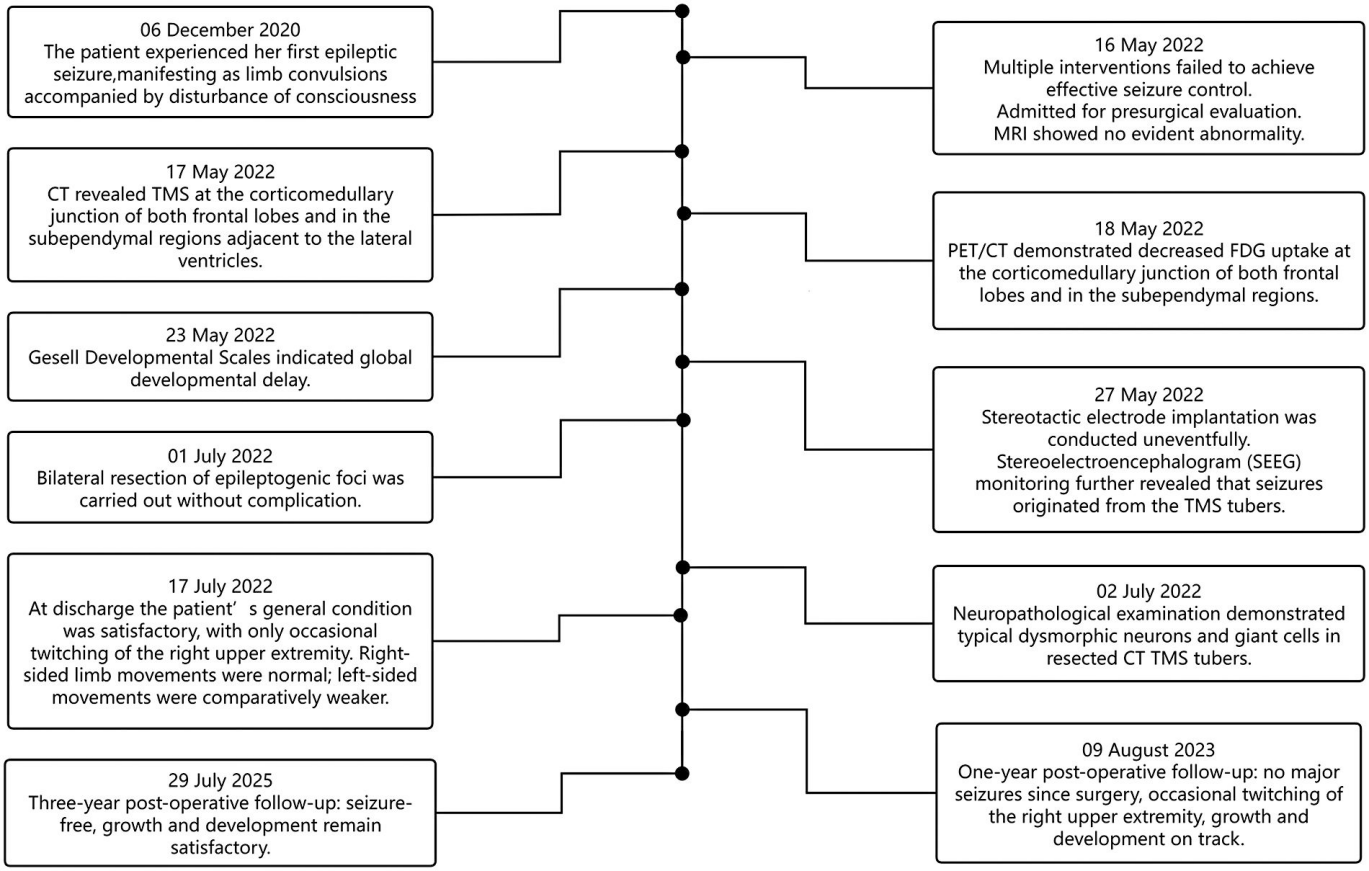
Patient timeline. MRI, magnetic resonance imaging; PET, positron emission tomography; CT, computed tomography; TMS, transmantle sign; FDG, fluorodeoxyglucose; T, temperature.

The patient's clinical timeline is shown in Figure 1. A 2-year-old girl presented with her first epileptic seizure at the age of 6 months, characterized by limb convulsions accompanied by disturbance of consciousness, which prompted her initial hospitalization. Subsequently, the seizures gradually progressed to daily focal seizures. The patient was allergic to oxcarbazepine. Despite sequential treatment with topiramate, corticosteroid pulse therapy, and a combination of vigabatrin and rapamycin, seizure control was not achieved. Following a 1.5-year clinical course of refractory epilepsy, she was admitted to our department for the evaluation of epilepsy surgery. A 3-year postoperative follow-up confirmed effective seizure controlled.

## 3 Diagnostic assessment

### 3.1 Clinical assessment

**Figure 2:**
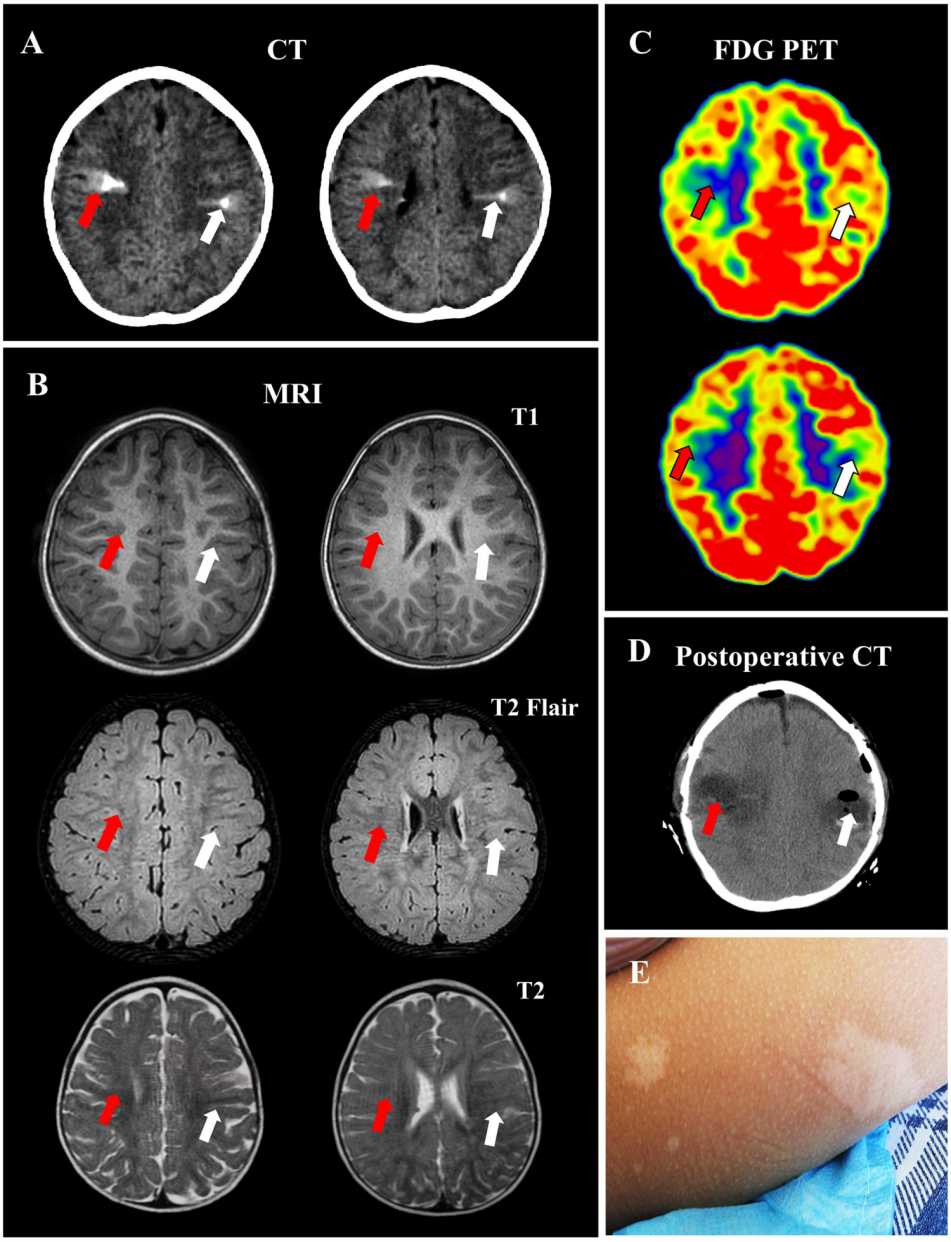
CT calcified radial lesion resembling TMS in the patient with TSC. Preoperative evaluation revealed high-density goblet-like CT lesions resembling TMS (arrows) **(A)** No significant hyperintensities were observed on T1, T2 Flair and T2 images from MRI (arrows) **(B)** Hypointense lesions were identified on FDG PET images (arrows) **(C)** Postoperative image shows the CT calcified radial cortical tuber were resected **(D)** Hypopigmentation was noted on the patient's skin **(E)** TMS, transmantle sign; TSC, tuberous sclerosis complex; FLAIR, fluid-attenuated inversion recovery; MRI, magnetic resonance imaging.

Physical examination revealed multiple 1–3 cm hypopigmented macules scattered over the head, back, and lower limbs. The macules exhibited a uniform pale white color, contrasting with the surrounding normal skin, and had relatively clear boundaries (Figure 2). Hypopigmented macules are major cutaneous signs of TSC and typically appear at birth or within the first few months of life. The patient was a premature infant with history of neonatal hypoxia. Her parents were healthy and non-consanguineous. Prior to disease onset, her intellectual development lagged behind age-matched peers, and the Gesell Developmental Schedules showed a significantly reduced developmental quotient (DQ), confirming developmental delay (Table 1).

**Table 1:** Gesell developmental schedules conducted for this patient.

Variables	Developmental assessment	Values
Gross Motor Behavior	Developmental Age (month)	12.4
	Developmental Quotient (%)	54
Fine Motor Behavior	Developmental Age (month)	5.2
	Developmental Quotient (%)	22
Adaptive Behavior	Developmental Age (month)	5.0
	Developmental Quotient (%)	22
Language Behavior	Developmental Age (month)	7.9
	Developmental Quotient (%)	34
Personal-Social Behavior	Developmental Age (month)	6.1
	Developmental Quotient (%)	26

### 3.2 Imaging examinations

A 3.0 T MRI scanner (Philips) was used with sequence parameters as follows: T1-weighted 3D MPRAGE (TR = 6.47 ms, TE = 3.07 ms, TI = 850 ms, slice thickness = 1.5 mm) and T2-FLAIR (TR = 4,800, TE = 160, TI = 2,000, slice thickness = 1 mm). Cranial CT revealed distinct bilateral hyperdense lesions with a goblet-like appearance in the central cerebral regions, resembling TMS (Figure 2A). Cranial MRI revealed no obvious T1 hypointensitie or T2-FLAIR hyperintensities (Figure 2B). TMS is typically characterized by T2-FLAIR hyperintensities in the subcortical white matter, tapering from the gray–white matter junction toward the lateral ventricle. Although TMS is considered a typical MRI feature of focal cortical dysplasia (FCD) type II, the CT-detected hyperdense radial lesion, morphologically similar to the TMS on MRI, is extremely rare.

Epi foci typically exhibit hypometabolism during the interictal period, appearing as hypointense regions on ^18^F-FDG PET/CT. In this patient, the CT calcified radial lesions resembling TMS showed hypometabolism (Figure 2C), suggesting these regions were potential epi foci. Additionally, subependymal nodules (SENs) with partial calcification, common cerebral imaging features of TSC, were observed in the right lateral ventricle on both CT and MRI. Taken together, the clinical manifestations and imaging findings supported a TSC diagnosis, with CT hyperdense lesions considered potential epi foci. 

### 3.3 Electroencephalogram (EEG) monitoring

**Figure 3:**
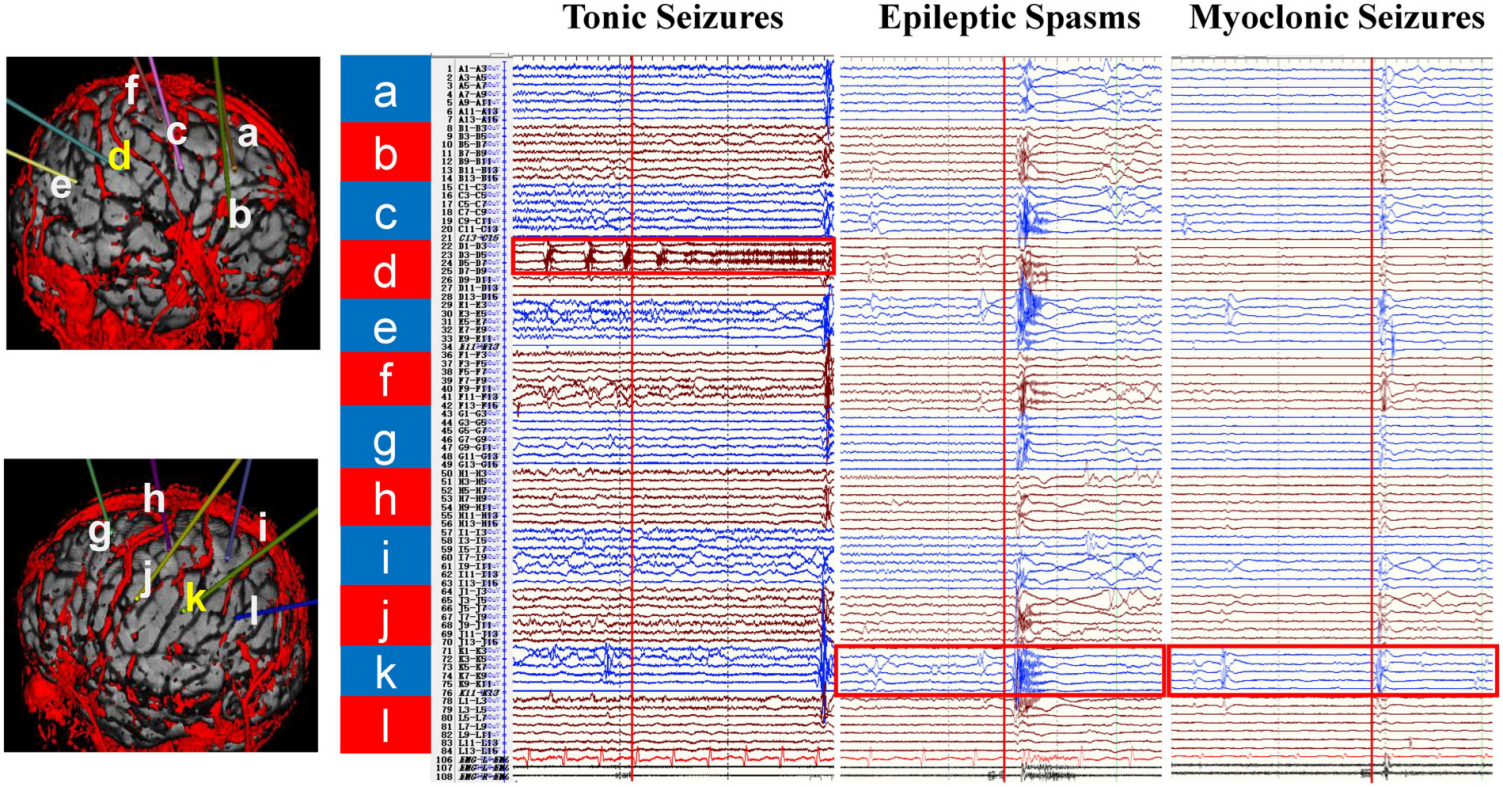
SEEG monitoring results in patients with TSC. Electrodes a-l were implanted in bilateral cerebral hemispheres **(a–f)** right hemisphere; **(g–l)** left hemisphere), covering the CT calcified radial lesion resembling TMS and their adjacent cortices. SEEG monitoring showed focal tonic seizures originating from the right lesion (electrode d), and focal epileptic spasms and myoclonic seizures originating from the left lesion (electrode k). SEEG, Stereoelectroencephalogram; TSC, tuberous sclerosis complex; TMS, transmantle sign.

To accurately localize the epi foci and confirm the diagnosis, stereotactic electrode implantation and long-term electroencephalogram (EEG) monitoring were performed. Scalp video EEG (VEEG) showed three types of focal epileptic seizures, including tonic seizures, epileptic spasms, and myoclonic seizures. Stereoelectroencephalogram (SEEG) monitoring was performed to refine seizure localization. Twelve electrodes (labelled a-l) were implanted in the bilateral cerebral hemispheres, with electrodes a-f in the right hemisphere and g-l in the left hemisphere. These electrodes covered the CT calcified lesions and adjacent cortical areas. The purpose of electrode placement was to record the electrical activity of the suspected epi foci, monitor adjacent cortical regions and exclude their involvement in seizure origination, and differentiate the origin sites of the three seizure types. SEEG monitoring confirmed that focal tonic seizures originated from the right CT calcified lesion (electrode d), while focal epileptic spasms and myoclonic seizures originated from the left CT calcified lesion (electrode k) (Figure 3).

### 3.4 Genetic testing

**Figure 4:**
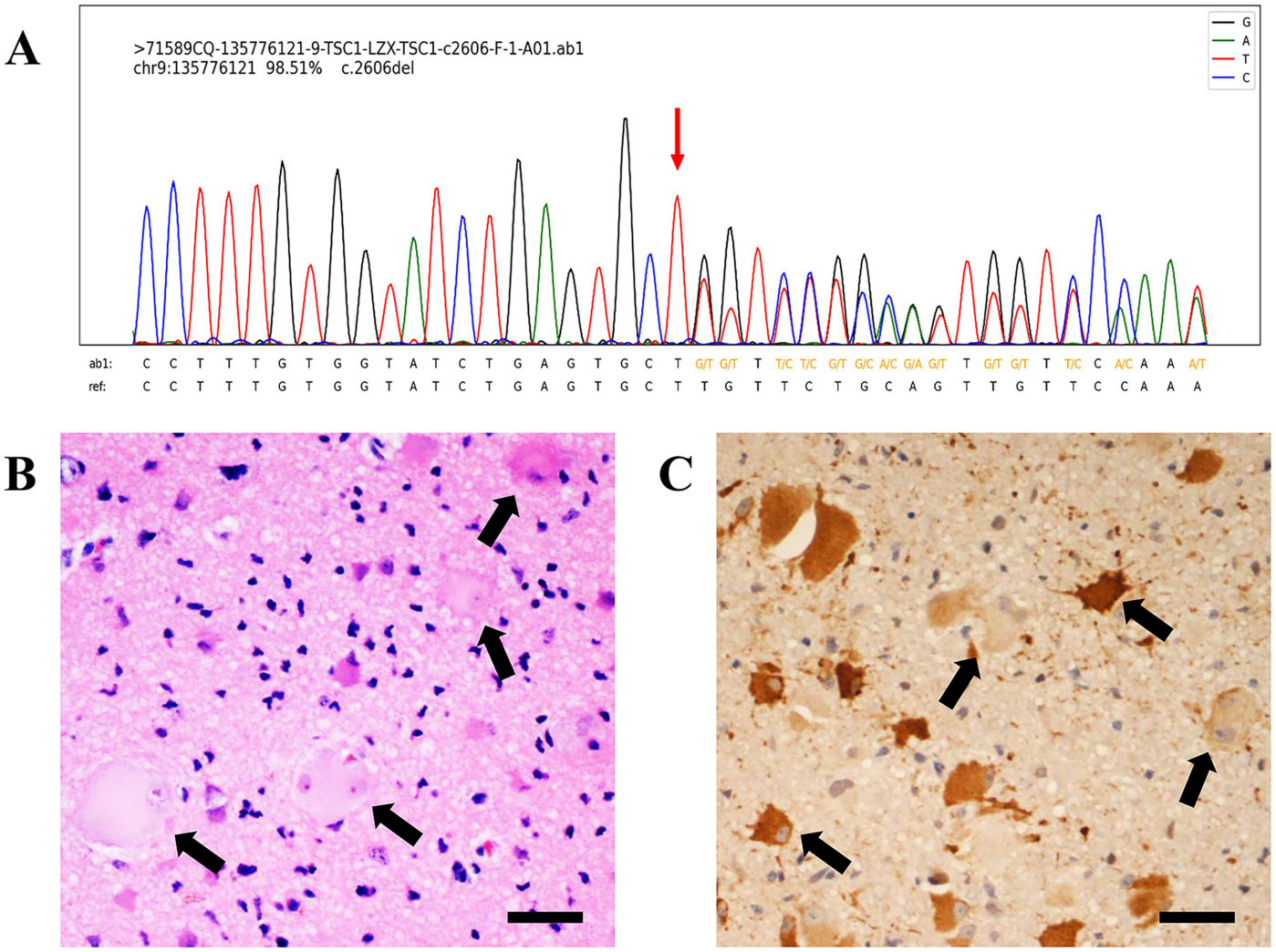
Sanger sequencing chromatogram of genetic testing and pathological examination in patients with TSC. Genetic testing results confirmed a heterozygous mutation (c.2606del) in TSC1 **(A)** Both HE staining **(B)** and immunohistochemical staining **(C)** demonstrate that the lesional area contains numerous balloon cells and dysmorphic neurons (arrows). HE, hematoxylin-eosin; immunohistochemical imaging was stained with vimentin, scale bar = 50 μm.

We employed clinical exome sequencing with standard blood samples to confirm the genetic mutation. Genetic analysis confirmed a heterozygous mutation (c.2606del) in TSC1, further confirming the diagnosis of TSC. A high-resolution Sanger sequencing chromatogram shows the presence of the genetic mutation (Figure 4A).

### 3.5 Neuropathological examination

Based on the clinical assessment, imaging examinations, EEG monitoring, and genetic analysis, the bilateral CT calcified radial lesions resembling TMS were considered epi foci and subsequently resected. Postoperative images confirmed resection of the lesions (Figure 2D). Neuropathological examination revealed abundant dysmorphic neurons and giant cells, which are pathological biomarkers of epi foci (Figures 4B, C).

### 3.6 Follow-up results

A 3-year postoperative follow-up confirmed effective seizures control (Table 2), showing that both resected bilateral CT calcified radial lesions resembling TMS were epi foci.

**Table 2:** Observations conducted in the postoperative follow-up.

Observation	1-year follow-up	2-year follow-up	3-year follow-up
Surgical outcomes	ILAE Class 1	ILAE Class 1	ILAE Class 1
scalp EEG monitoring	no significant epileptiform discharges	no significant epileptiform discharges	no significant epileptiform discharges
Seizure frequency	—	—	—
Seizure type	—	—	—
Numbers of ASMs	2	2	2
Developmental assessment	severe developmental delay	moderate developmental delay	moderate developmental delay

ASMs, antiseizure medications.

## 4 Discussion

This report describes a rare case of TSC with no clear cortical tubers on MRI but with the CT calcified radial lesions resembling TMS. Conventional MRI did not reveal clear cortical tubers, while CT identified rare bilateral hyperdense TMS-like lesions in the central brain regions. This case highlights the clinical value of CT in identifying epi tubers, particularly when cortical tubers are atypical on MRI. CT-specific features, including TMS-like lesions, offer unique imaging clues for identifying epi tubers and provides strong evidence to support surgical intervention.

TSC is an autosomal dominant genetic syndrome with an incidence of 1 in 6,000–22,000 live births. Over 80% of patients experience epileptic seizures, and approximately 80% of these cases are diagnosed before two years of age, often presenting with infantile spasms or focal seizures. More than 54% of patients with TSC experience DRE, and persistent seizures are associated with an increased risk of intellectual disability (3, 5). DRE in TSC poses a significant challenge in clinical practice, and surgical treatment remains an effective therapeutic option. Multiple cortical tubers are a typical manifestation of TSC, and are the origin of epileptogenesis (12). The precise preoperative identification of epi tubers from all cortical tubers is particularly critical for TSC-related epileptic surgery (5, 13, 14).

Neuroimaging is widely used to evaluate brain function and detect abnormalities in various neurological disorders, offering high sensitivity, specificity, and accuracy for detecting foci. Advances in neuroimaging and data postprocessing methods have improved delineation of epi foci (15), and are indispensable noninvasive approaches for preoperative evaluation of TSC (16, 17). The imaging spectrum of neurological lesions in TSC presents a biological trajectory from dysplasia and hamartomas to tumor formation. MRI is typically critical for characterizing these lesions. Conventional MRI is the first choice for presurgical assessment, helping to understand the neurobehavioral characteristics of multiple cortical tubers. Cortical tubers appear as well-defined hyperintensities on T2-FLAIR sequences, with T1 hypointense areas often extending from the cortical surface into the white matter. SEN presents hyperdense calcifications on CT, isointense or slightly hyperintense on T1-weighted images and hypointense on T2-weighted images. Enhancement of tubers or a diameter exceeding 5 mm should raise suspicion of transformation into SEGA, which exhibits homogeneous or tuber enhancement, increased perfusion on ASL, and no diffusion restriction on DWI, allowing preoperative differentiation from high-grade gliomas. Radial white matter migration tracts can be visualized as 1–2 mm linear hyperintensities on T2-FLAIR, extending from the ependymal surface to the cortical tubers (18, 19).

Notably, 5%–10% of TSC cases have atypical cortical tubers on MRI, and these cortical tubers are traditionally identified via metabolic abnormalities on MRS or PET (20, 21). Prior literature highlights CT's utility for detecting calcified subependymal nodules or occasional low-density cortical lesions in such cases (22, 23), while solitary hyperdense cortical lesions on CT are rare and prone to misdiagnosis (24). In this case, MRI did not reveal the typical features of cortical tubers, but CT identified rare bilateral hyperdense TMS-like lesions in the central brain regions, corroborated by clinical features and genetic testing. These lesions were distinct from calcified subependymal nodules or occasional low-density cortical lesions previously described. This finding reinforces that for patients with TSC but inconspicuous MRI findings, CT may identify calcifications and hyperdense TMS-like lesions corresponding to pathologically validated epi tubers, refining diagnostic workflows.

TMS is a typical imaging criterion used to identify focal cortical dysplasia IIb (FCDIIb) on MRI (1, 2). Balloon cells are considered the pathological basis of TMS. Isolated dysmorphic neurons are insufficient to induce TMS, whereas the interspersion of balloon cells among dysmorphic neurons can elicit typical TMS (25). Therefore, among patients with FCD, those with type IIb FCD with a large number of balloon cells are more likely to exhibit TMS (25). Notably, balloon cells are accompanied by large, dystrophic glioneuronal components. Furthermore, gliosis within these components and high balloon cell density may be associated with calcification on CT (26). TSC and FCDIIb are typical malformations of cortical development with similar clinical and pathological characteristics. Balloon cells are also a typical neurophysiological feature of TSC, suggesting that TMS may be the key indicator for diagnosing TSC (4). Calcification is a clue for identifying epi tubers and is usually confined to the grey matter (3). Both our findings and those of other researchers have confirmed that outstanding cortical tubers measuring > 3–4 cm and containing a nidus of calcification carry a higher risk of developing into epi tubers (3, 27). These results further highlight the epileptogenic potential of cortical tubers with calcification. However, CT calcified radial lesion resembling TMS presents as calcifications in the deep white matter and grey-white matter junction, which are extremely rare. Although the presence of CT calcified radial lesion resembling TMS has not yet been included in the diagnostic criteria for TSC, it demonstrates unique value in identifying epi tubers and provides strong evidence supporting surgical intervention. Therefore, despite its rarity, it is often crucial for determining epi foci.

Based on this case, brain MRI remains the diagnostic test of choice, and any supplementation with CT should be adequately evaluated in specific cases as part of a comprehensive diagnostic workup that also includes multimodal neuroimaging. Multimodal medical imaging combining information from various imaging modalities could provide a comprehensive understanding of epi foci in clinical diagnosis and research and integrate the location of abnormal foci on a patient-specific brain map. Multimodal image integration shows great superiority in helping delineate epi foci, further confirming the value of imaging clues in identifying epi foci.

## 5 Conclusion

Precise preoperative localization of epi tubers among cortical tubers is key to determining the resection approach, surgical outcomes, and prognosis of TSC. This case describes a rare finding in a 2-year-old female patient with TSC and bilateral CT-detected calcified radial lesions in the central cerebral regions resembling TMS, without corresponding findings on MRI. The patient underwent bilateral resection of the CT calcified radial tubers. Comprehensive preoperative evaluation, including scalp and intraoperative EEG monitoring, neuropathological examination, and a 3-year postoperative follow-up, confirmed that both resected tubers were epi tubers. Our study highlights that CT calcified radial lesion resembling TMS is a value imaging feature for accurately identifying epi tuber and guiding surgical planning. While MRI remains the first-line modality for evaluating TSC-related epi tubers, TMS-like calcifications on CT serve as important clues for their localization. Clinical implications of multimodal imaging for epileptogenic foci localization in TSC patients with subtle MRI findings is important. Identification of the CT calcified radial lesion resembling TMS has important clinical implications for optimizing the surgical management of TSC-associated DRE.

## 6 Patient perspective

The patient's mother provided the following clinical perspective: Our daughter's first seizure occurred at 6 months of age, initiating a 1.5-year course of refractory daily focal seizures. Multimodal therapies failed to control the seizures. We noted clear motor and language developmental delays relative to peers. Epilepsy surgery served as the only viable option. Postoperatively, the seizures ceased completely, accompanied by progressive developmental gains, for which we are profoundly grateful.

## Data Availability

The original contributions presented in the study are included in the article/Supplementary Material, further inquiries can be directed to the corresponding author.
